# Selective Inhibition of Murine Cytomegalovirus Viral Gene Expression by the Antiviral Peptide TAT-I24

**DOI:** 10.3390/ijms23137246

**Published:** 2022-06-29

**Authors:** Hanna Harant

**Affiliations:** Pivaris BioScience GmbH, 1030 Vienna, Austria; hanna.harant@pivaris-bioscience.at

**Keywords:** antiviral peptide, cell-penetrating peptide, murine cytomegalovirus, viral gene expression, drug combinations

## Abstract

The effect of the antiviral peptide TAT-I24 on viral gene expression in cells infected with murine cytomegalovirus (MCMV) was investigated. The expression of immediate-early, early and late genes was highly induced upon infection with MCMV. In the presence of the peptide, the expression of all tested genes was sustainably reduced to a similar extent, independent of whether they were immediate-early, early or late genes. In contrast, the expression of host genes, such as NF-κB inhibitor alpha (*Nfkbia*), interferon-induced protein with tetratricopeptide repeats 1 (*Ifit1*), chemokine (C-X-C motif) ligand 10 (*Cxcl10*), chemokine (C-C motif) ligand 7 (*Ccl7*) and chemokine (C-C motif) ligand 5 (*Ccl5*), which are induced early upon virus infection, was only transiently suppressed in peptide-treated cells. The expression of other host genes which are affected by MCMV infection and play a role in endoplasmic reticulum stress or DNA-damage repair was not inhibited by the peptide. A combination of TAT-I24 with the nucleoside analogue cidofovir showed enhancement of the antiviral effect, demonstrating that viral replication can be more efficiently inhibited with a combination of drugs acting at different stages of the viral life-cycle.

## 1. Introduction

At present, only a limited number of approved antiviral drugs are available, and many of those are only suitable for the treatment of infections by a specific virus [1,2,3,4]. As such, broad-spectrum antiviral drugs are urgently needed to target several different viruses and manage infections by known and newly emerging viruses. The lack of suitable broad-acting drugs was dramatically outlined by the SARS-CoV-2 pandemic [5]. Besides small molecules, which can interfere with virus replication, such as nucleoside analogues [6] or other drugs which target viral enzymes such as proteases [3], several antiviral peptides (AVP) have been identified, mostly entry/fusion inhibitors [7,8]. Two antiviral peptides are available on the market: enfuvirtide (Fuzeon^®^), for the treatment of infections by human immunodeficiency virus-1 [9], and bulevirtide (Hepcludex^®^), for the treatment of chronic hepatitis D infections [10]. However, there are still limitations with the use of peptide drugs for the treatment of infectious diseases, including their poor serum stability, degradation by proteases, lack of bioavailability and lower potencies [11]. Some of these issues can be overcome by modifying the peptides, such as by conjugation to polyethylene glycol, which leads to enhanced stability. Other approaches include the use of non-natural amino-acid residues, substitution of l- by d-amino acids or cyclization [12,13,14]. 

We have described the novel antiviral peptide TAT-I24, a fusion of the 9-mer peptide I24 and the TAT peptide, which exerts broad-spectrum activity against several double-stranded DNA viruses [15]. The peptide is able to inhibit replication of herpes simplex virus, human and mouse cytomegalovirus (CMV), vaccinia virus, some adenoviruses and SV40 polyomavirus. Partial inhibition was also observed with human immunodeficiency virus-1 (HIV-1) and respiratory syncytial virus (RSV), indicating that the inhibitory effect is not restricted to viruses with double-stranded DNA genomes [15]. The antiviral activity is caused by an effect on virus entry and by binding to the viral nucleic acid, leading to a blockade of viral gene expression, exemplified by a reduction of reporter gene expression from a non-replicating baculovirus in mammalian cells [15]. The direct interaction of TAT-I24 with the viral genome is also evident from the co-localization of fluorescently labelled peptide and murine cytomegalovirus (MCMV) DNA early after infection; this is further supported by the ability of the peptide to bind double-stranded DNA with high affinity [15,16]. These studies indicated selective inhibition of viral gene expression with little or no effect on host-cell gene expression 24 to 72 h post-infection. However, it remained to be elucidated how TAT-I24 affects the cascade of early and late viral gene expression as well as the gene expression of the host cell in response to the infection. The present study therefore addressed the effect of TAT-I24 on viral and host cell gene expression at early phases of infection with MCMV.

## 2. Results

### 2.1. Dose-Dependent Antiviral Activity of TAT-I24 against MCMV

**Figure 1:**
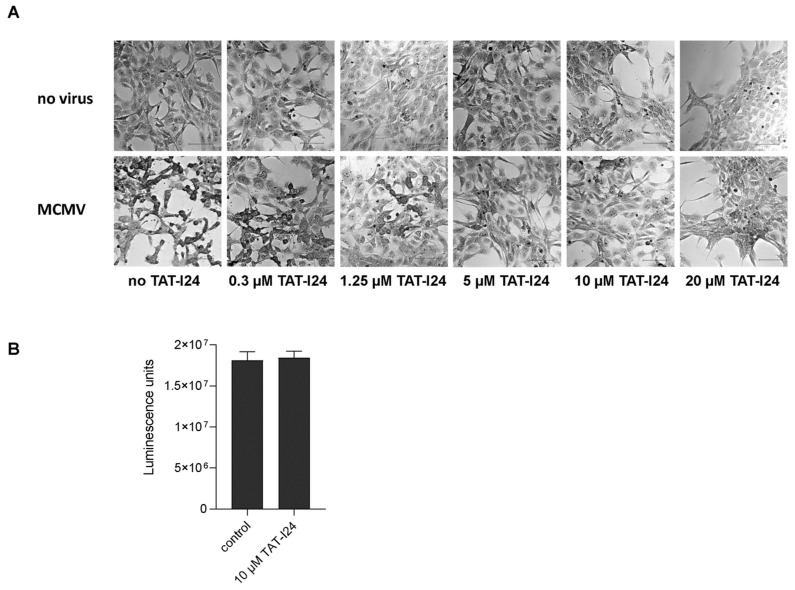
Crystal violet staining of uninfected NIH/3T3 cells and cells infected with MCMV (MOI 1) in the presence of increasing concentrations of TAT-I24 48 h post-infection (Differential Interference Contrast (DIC); 20× objective). Scale bars indicate 100 µm (**A**). Viability of vehicle-treated cells (control) and cells treated with 10 µM TAT-I24 for 48 h. Results shown are mean ± standard deviation of eight replicates per group (**B**).

In our previous reports, we demonstrated that peptide TAT-I24 exerts antiviral activity against MCMV expressing luciferase [15,16]. This strain was also used in the present investigation. Crystal violet staining of NIH/3T3 cells infected with MCMV (multiplicity of infection; MOI 1) showed characteristic cytopathic effects 48 h post-infection (Figure 1A). However, with increasing concentrations of TAT-I24, the cytopathic effects caused by the virus infection were inhibited (Figure 1A). At 20 µM, TAT-I24 has some effect on cell attachment and morphology but does not affect cell viability, as previously reported [15]. For all further investigations, 10 µM of TAT-I24 was therefore chosen to ensure efficient inhibition of MCMV infection. By using a cell viability assay, it was further confirmed that the peptide does not exert any cytotoxic effect at this concentration (Figure 1B).

### 2.2. TAT-I24 Inhibits MCMV Viral Gene Expression at Early Time-Points of Infection

After infection with human CMV (HCMV), a cascade of immediate-early, early and late gene expression is initiated [17,18,19]. Immediate early (IE) genes are required for viral gene transcription before the onset of replication and for the inhibition of the innate host cell response to infection. Early genes are required for viral DNA replication and packaging, while late genes encode structural proteins and tegument after DNA replication [20]. Murine cytomegalovirus (MCMV) shares many features of HCMV and is therefore widely accepted as a model for CMV infections. Also, the HCMV and MCMV genome structures are similar [21]. Due to these features and its similarity with HCMV, this virus was chosen to investigate early events of viral gene transcription in the absence and presence of the antiviral peptide TAT-I24. 

NIH/3T3 fibroblasts were infected with MCMV expressing luciferase at low MOI (MOI 1) with the aid of centrifugal enhancement. Infections were performed either in the absence or presence of 10 µM TAT-I24. Cells were lysed after 30 min or 2-, 4-, 8- and 24-h post-infection. Total RNA was isolated and treated with DNase I to remove viral DNA before synthesis of the cDNA. Levels of viral transcripts were then analyzed by quantitative PCR and normalized to mRNA levels of the glyceraldehyde-3-phosphate dehydrogenase (*Gapdh*) housekeeping gene.

**Figure 2:**
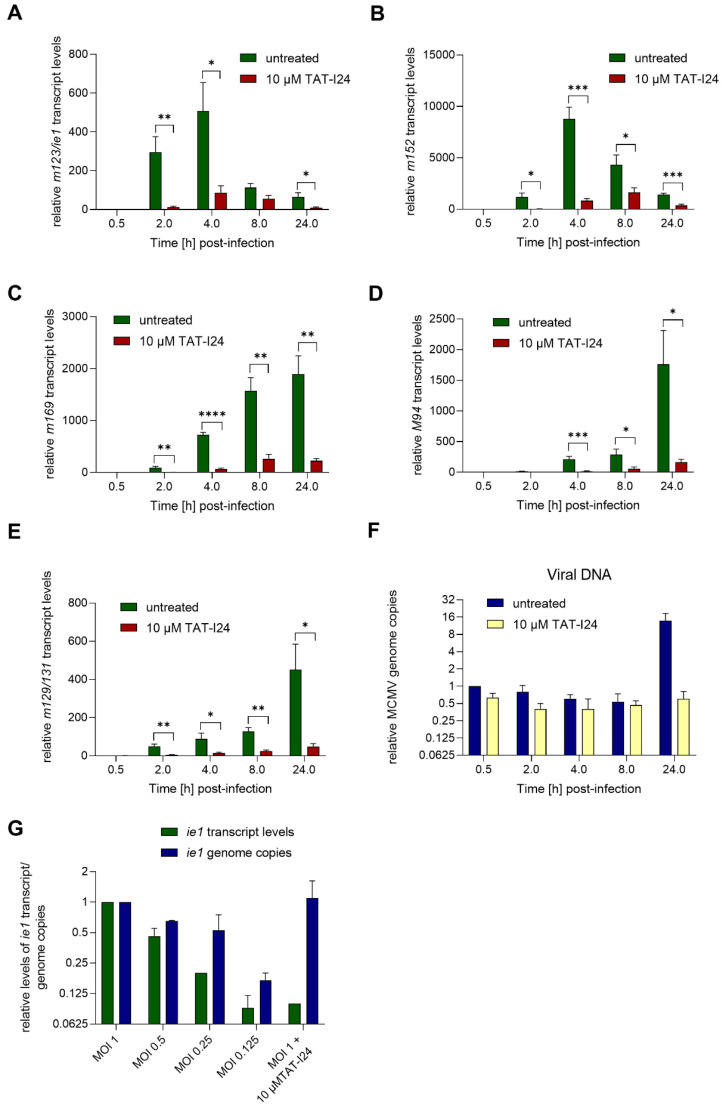
MCMV viral gene transcript levels relative to the housekeeping gene *Gapdh* in NIH/3T3 cells infected with MCMV (MOI 1) in the absence or presence of 10 µM TAT-I24. Fold change of transcript levels (mean ± standard error of the mean) from four independent experiments is shown (**A**, *m123/ie1*; **B**, *m152*; **C**, *m169*; **D**, *M94*; **E**, *m129/131*). Multiple t-test was used for statistical analysis; * statistically significant at *p* ≤ 0.05; ** statistically significant at *p* ≤ 0.01, *** statistically significant at *p* ≤ 0.001, **** statistically significant at *p* ≤ 0.0001. MCMV viral DNA determined by *ie1* PCR relative to the 30 min control (**F**). Transcript levels of *ie1* or viral genome copies relative to MOI 1 determined by *ie1* PCR from cells infected at various MOI or MOI 1 with 10 µM TAT-I24 2 h post-infection. Mean ± standard error of the mean from three (**F**) or two independent experiments is shown (**G**).

During lytic infection with MCMV, transcription of immediate-early (IE) genes *ie1* and *ie3* is controlled by the major immediate early promoter/enhancer (MIEP) and involves differential splicing [22,23]. MIEP contains binding sites for host-cell transcription factors, such as nuclear factor-κB (NF-κB) [24], interferon regulatory factor-1 and virion-associated factors [25]. The IE1 protein can cause transactivation of viral and cellular genes and localizes to specific sites in the nucleus, i.e., nuclear domain 10 (ND10), where active viral gene transcription is ongoing [26]. In the present setting, the expression of the immediate early gene *ie1* was induced 2 h after infection with MCMV, continued to rise until 4 h post-infection and declined afterwards, as seen by reduced levels 8 h post-infection. This observed kinetics of *ie1* gene expression was in accordance with the study by Marcinowski et al., 2012 [27]. However, when infection was performed in the presence of 10 µM TAT-I24, overall levels of *ie1* transcripts were strongly reduced, i.e., by more than 90% compared to untreated cells 2 h post-infection, and similar inhibition was seen at later time points (Figure 2A). 

A similar picture was obtained with the early gene *m152*, encoding the glycoprotein gp40, the expression of which is highly induced upon MCMV infection 4 h post-infection and declining 8 h post-infection. As with *ie1* transcripts, the levels of *m152* transcripts were also strongly reduced in the presence of 10 µM TAT-I24 (Figure 2A,B). Another early gene is *m169*, the expression of which starts to rise significantly after 4 h, with transcript levels continuously increasing thereafter. Again, although *m169* gene expression increased with the same kinetics in peptide-treated cells, mRNA levels were strongly reduced in the presence of TAT-I24 (Figure 2C). 

One of the late MCMV genes, *M94*, was also included in this analysis. Levels of *M94* transcripts started to increase after 4 h and continued to rise until 24 h, the latest time-point tested. Again, gene expression of *M94* was strongly inhibited by TAT-I24 (Figure 2D). Similar effects were seen with the spliced late gene *m129/131* [27,28], the expression of which was reduced by the presence of TAT-I24. 

The levels of viral DNA in the RNA extracts were also determined by PCR using the *ie1* primer pair. In peptide-treated cells, the amount of viral DNA was about 60% compared to untreated cells. The amount of viral DNA declined in both settings between 2 and 8 h. However, after 24 h, where replication of the viral genome starts, increase in viral DNA was observed in untreated cells while it remained at background levels in the presence of TAT-I24 (Figure 2F). This is in accordance with our earlier study using detection of the luciferase gene of MCMV strain delm157-luc rep [16]. These data show that independent of whether a MCMV gene belongs to the immediate-early, early or late genes, transcript levels are strongly reduced in the presence of TAT-I24.

Earlier studies showed that the peptide can inhibit viral entry, but that it also binds DNA with high affinity, leading to a block of viral gene transcription [15,16]. To evaluate whether the inhibition of *ie1* transcript levels could be due to reduced virus entry, NIH/3T3 cells were infected with MCMV at various MOI or with MOI 1 in the presence of 10 µM TAT-I24. After 2 h, RNA was isolated and *ie1* transcript levels and genome copies analyzed by quantitative PCR. Levels of *ie1* transcripts were normalized to the *Gapdh* housekeeping gene. As expected, *ie1* transcript levels decreased, with a reduction of the infectious particles, a finding reflected by reduced genome copies. In contrast, cells infected with the highest dose of MCMV in the presence of 10 µM TAT-I24 showed reduction of *ie1* transcript levels by 90%, but this was not reflected at the level of viral DNA (Figure 2G). These findings further support the notion that the inhibitory effect of TAT-I24 is mediated by direct suppression of viral gene expression through interaction of the peptide with the viral DNA. 

### 2.3. TAT-I24 Transiently Represses Expression of Genes Induced upon MCMV Infection

**Figure 3:**
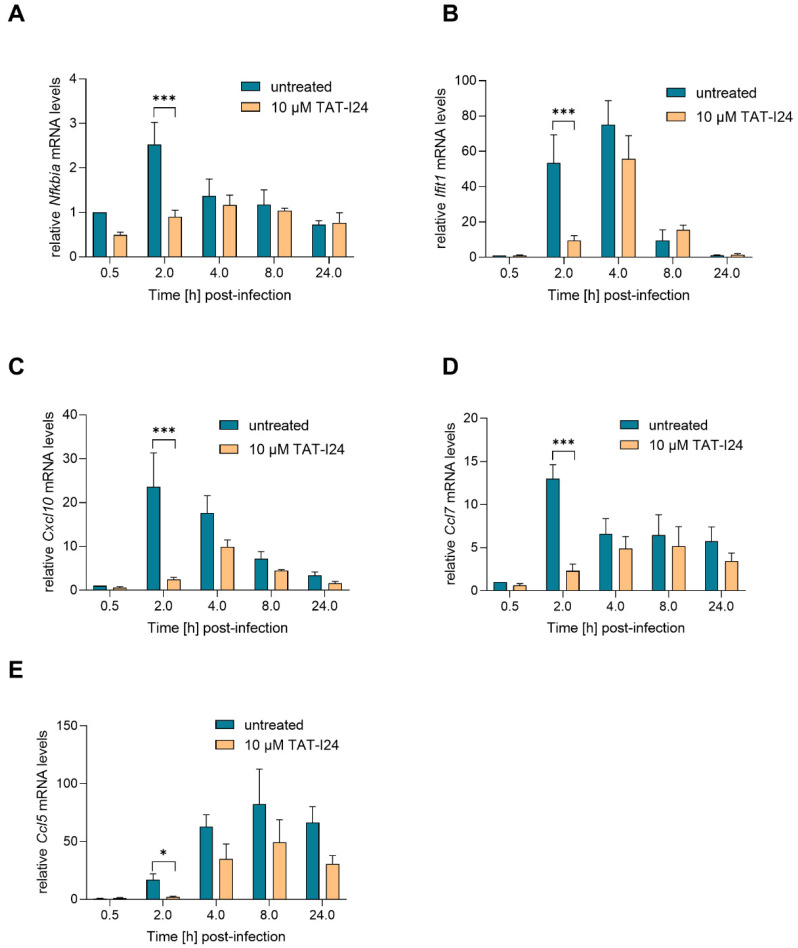
Levels of host-cell transcripts relative to the housekeeping gene *Gapdh* (**A**, *Nfkbia*; **B**, *Ifit1*; **C**, *Cxcl10*; **D**, *Ccl7*; **E**, *Ccl5*) in NIH/3T3 cells infected with MCMV (MOI 1) in the presence or absence of 10 µM TAT-I24. Fold change in transcripts levels (mean ± standard error of the mean) from four independent experiments is shown. Multiple t-test was used for statistical analysis; * statistically significant at *p* ≤ 0.05, *** statistically significant at *p* ≤ 0.001.

Activation of NF-κB is one of the earliest events during HCMV and MCMV infection, and this transcription factor complex can activate expression of immediate early-genes through binding to MIEP [25,29,30]. However, at later time-points, NF-κB activation is counter-regulated by the virus [31]. The IE1 protein itself has been shown to transactivate the NF-κB p105/50 promoter [24]. One NF-κB-responsive gene is NF-κB inhibitor alpha (*Nfkbia*), which is upregulated upon MCMV infection [27,31]. *Nfkbia* transcript levels were upregulated by infection with MCMV, peaking at 2 h and then declining to background levels. However, 2 h post-infection, *Nfkbia* transcript levels were also reduced when infection occurred in the presence of TAT-I24 (Figure 3A). Four hours post-infection, the levels of *Nfkbia* mRNA were comparable between untreated and peptide-treated cells. 

Upon infection with HCMV or MCMV, the viral DNA is recognized by cytosolic DNA sensors, leading to type-I interferon production through activation of interferon-regulatory factor-3 (IRF3). In addition, an interferon-independent pathway involves activation of IRF-3 and interferon-stimulated gene (ISG) regulation. These include ISG15, virus inhibitory protein, endoplasmic reticulum-associated, IFN-inducible (viperin), interferon-induced protein with tetratricopeptide repeats (IFIT) proteins, interferon-inducible transmembrane (IFITM) proteins as well as myxovirus resistance proteins A and B [32]. A strong upregulation of *Ifit1* mRNA expression upon infection with MCMV was shown in the study by Marcinowski et al. [27]. In the present study, strong up-regulation of *Ifit1* transcript levels was also observed upon infection with MCMV, an effect which was significantly reduced by TAT-I24 2 h post-infection. However, after 4 h, *Ifit1* mRNA levels also increased in the presence of the peptide, followed by a decline after 8 h in both settings (Figure 3B). This indicates that the expression of these host genes is delayed rather than inhibited by the presence of TAT-I24. 

The expression of several chemokines is also upregulated during MCMV infection, such as that of chemokine (C-X-C motif) ligand 10 (Cxcl10; also known as interferon inducible protein 10/IP-10), chemokine (C-C motif) ligand 7 (Ccl7; also known as MCP-3) and chemokine (C-C motif) ligand 5 (Ccl5; also known as RANTES) [27,31,33]. In the current setting, transcript levels of the chemokines *Cxcl10* and *Ccl7* were also rapidly upregulated upon MCMV infection and reduced by TAT-I24 2 h post-infection. However, at 4 h post-infection, levels of *Cxcl10* and *Ccl7* started to increase, also in the presence of TAT-I24 (Figure 3C,D). *Ccl5* transcript levels were low 2 h post-infection and increased 4 and 8 h after infection. Again, a reduction of *Ccl5* transcript levels in the presence of TAT-I24 was observed (Figure 3E).

**Figure 4:**
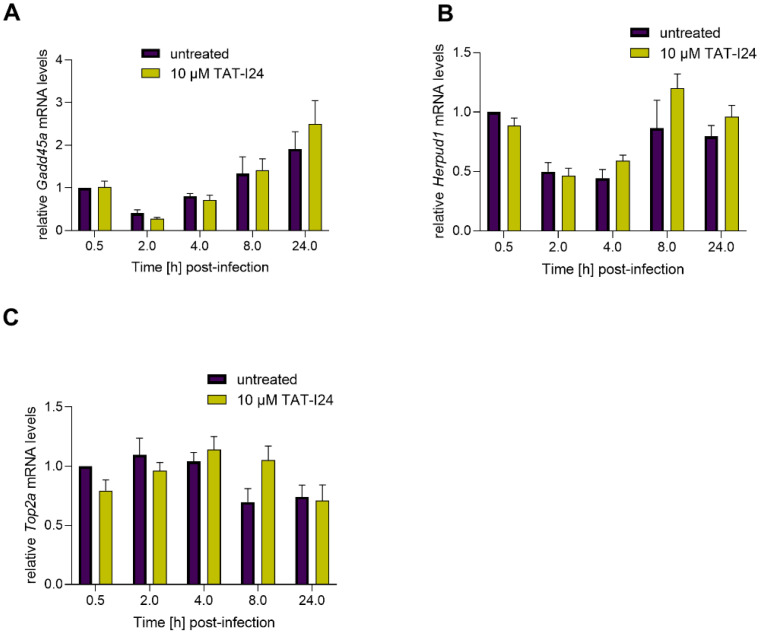
Levels of host-cell transcripts relative to the housekeeping gene *Gapdh* (**A**, *Gadd45a*; **B**, *Herpud1*; **C**, *Top2a*) in NIH/3T3 cells infected with MCMV (MOI 1) in the presence or absence of 10 µM TAT-I24. Fold change in transcripts levels (mean ± standard error of the mean) from four independent experiments is shown.

Based on the study by Marcinowski et al. 2012 on differential gene expression caused by MCMV infection compared to UV-inactivated virus [27], the expression of three host genes involved in DNA damage and endoplasmic reticulum stress (ER) were included in this study as well. Growth arrest and DNA damage-inducible protein A (*Gadd45a*), a DNA damage-induced gene [34], was found to be upregulated by MCMV infection [27]. In the current setting, we found a slight downregulation 2 h post-infection and a rise in transcript levels at later time-points. However, no difference in *Gadd45a* mRNA levels was seen between untreated and peptide-treated cells (Figure 4A). A similar effect was also seen with mRNA levels of Homocysteine-responsive endoplasmic reticulum-resident ubiquitin-like domain member 1 protein (*Herpud1*), a gene induced by ER stress [35]. Again, no differences in levels of *Herpud1* transcripts between untreated cells and peptide-treated cells were observed (Figure 4B). Topoisomerase 2 alpha (*Top2a*) [36], which has been shown to be downregulated by MCMV [27], was also slightly downregulated under the present conditions. However, no significant effect of TAT-I24 on *Top2a* transcripts levels was observed (Figure 4C). The regulation of the three genes in this study varies in their extent from the published data, but this difference is probably due to the lower MOI (MOI 1) used in this study compared to the MOI 10 used by Marcinowski et al. [27].

### 2.4. Reduction of Host-Cell Transcript Levels Is Dependent on DNA-Binding of the Peptide

**Figure 5:**
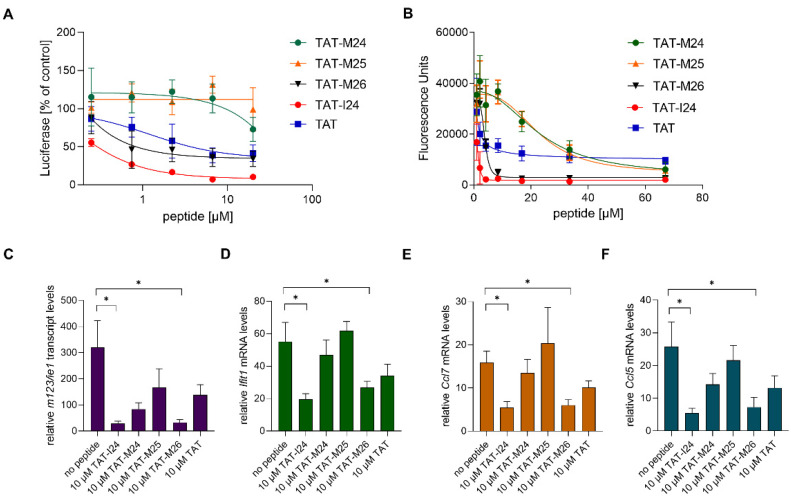
(**A**) Effect of peptides on luciferase reporter gene expression in NIH/3T3 cells infected with MCMV 72 h post-infection. Results shown are mean ± standard deviation from three independent experiments performed in duplicates for TAT-M24, TAT-M25 and TAT-M26. For comparison, TAT-I24 and TAT were included. (**B**) Fluorescence reduction of SYBR^®^ Gold-stained plasmid-DNA by various peptides. Results shown are mean ± standard deviation from three independent experiments performed in duplicates. (**C**–**F**) Levels of host-cell transcripts relative to the housekeeping gene *Gapdh* in NIH/3T3 cells infected with MCMV (MOI 1) 2 h post-infection. Results shown are fold change of transcript levels (mean ± standard error of the mean) of *m123/ie1* (**C**), *Ifit1* (**D**), *Ccl7* (**E**) and *Ccl5* (**F**) from four independent experiments. Multiple t-test was used for statistical analysis; * statistically significant at *p* ≤ 0.05.

TAT-I24 is a fusion of the DNA-binding TAT peptide and the 9-mer peptide I24 (CLAFYACFC) which also binds DNA. This fusion resulted in greatly enhanced affinity for DNA and generated a peptide with antiviral activity [15,16]. To determine whether reduction of host-gene expression early after MCMV infection is related to the ability of the peptide to bind DNA, variants of TAT-I24 were generated with modifications of the TAT fusion partner (Figure 5A). The peptides were tested for their potency in inhibiting MCMV replication and their binding to DNA. Peptide TAT-M26 contains six N-terminal amino-acid residues of the TAT peptide fused to I24 (GRKKRRCLAFYACFC), while TAT-M24 contains six C-terminal residues of the TAT peptide fused to I24 (RRRPPQCLAFYACFC). The peptide TAT-M25 only contains three amino-terminal arginine residues fused to I24 (RRRCLAFYACFC). DNA-binding analysis using a fluorescence-based assay [16] showed reduced DNA binding of peptides TAT-M24 and TAT-M25, while peptide TAT-M26 showed similar DNA binding as TAT-I24 (Figure 5B). The TAT peptide alone causes partial fluorescence reduction, as it also binds DNA (Figure 5B). The strongly reduced DNA binding activity of variants TAT-M24 and TAT-M25 also correlated with their lack of antiviral activity at the concentrations tested (Figure 5A). 

The peptide variants with reduced DNA binding were also less active in reducing *ie1* transcript levels 2 h post-infection, with only TAT-I24 and TAT-M26 exerting a significant inhibitory effect (Figure 5C). However, a partial reduction of *ie1* transcript levels was observed by the other peptides including the TAT peptide, which exerts a partial inhibitory effect on MCMV replication [15]. 

The peptides were also tested for their ability to cause reduction of host-gene expression induced upon infection with MCMV. Peptides TAT-I24 and TAT-M26, which were able to cause significant inhibition of *ie1* gene expression (Figure 5C), also caused a significant reduction of *Ifit1*, *Ccl7* and *Ccl5* expression 2 h post-infection (Figure 5D–F). 

**Figure 6:**
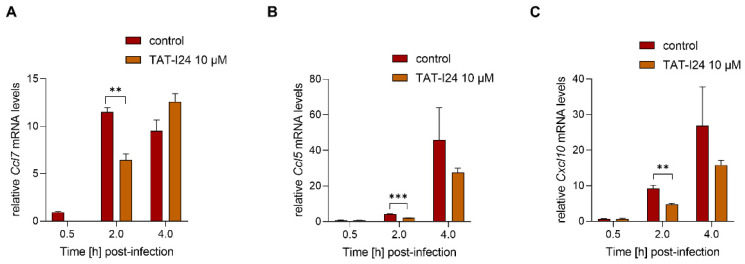
Levels of host-cell transcripts relative to the housekeeping gene *Gapdh* in cells infected with UV-irradiated MCMV (MOI 2.5) either untreated or treated with 10 µM TAT-I24. Fold change in transcripts levels (mean ± standard error of the mean) of *Ccl7* (**A**), *Ccl5* (**B**), or *CxCl10* (**C**) from three independent experiments is shown. Multiple t-test was used for statistical analysis; ** statistically significant at *p* ≤ 0.01, *** statistically significant at *p* ≤ 0.001.

To investigate a possible dependence of the reduction of host gene expression on inhibition of viral gene expression by TAT-I24, MCMV was inactivated by irradiation with ultraviolet (UV) light. NIH/3T3 cells were infected with UV-inactivated MCMV (MOI 2.5) before RNA isolation 30-min, 2- and 4-h post-infection. A higher MOI was required for the induction of sufficient transcript levels by the UV-inactivated virus. In this setting, TAT-I24 significantly reduced the transcript levels of *Ccl7*, *Cxcl10* and *Ccl5* 2 h post-infection, indicating that the inhibitory effect of the peptide is not linked to the reduction of viral gene expression (Figure 6A–C). 

**Figure 7:**
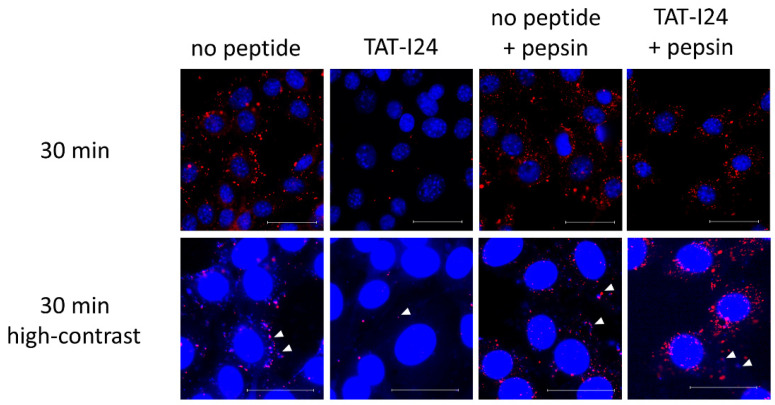
Localization of BrdU-labelled MCMV DNA in NIH/3T3 cells 30 min post-cold release. Cells were left untreated or incubated with TAT-I24 during MCMV adsorption. Cells were fixed and permeabilized before staining with BrdU-antibody (red fluorescence). Nuclei were additionally stained with DAPI (blue fluorescence). One group was treated with pepsin for 20 min after fixation and permeabilization followed by staining with the BrdU antibody. The lower panel shows enlarged images at high contrast settings (40× objective). Arrows indicate co-localization of blue fluorescence (DAPI) and red fluorescence of BrdU-labelled viral DNA. Scale bar indicates 40 µm.

When infection of cells is performed with MCMV labelled with bromodeoxyuridine (BrdU), a clear reduction in BrdU-labelled genomes in TAT-I24-treated cells can be observed [16]. It was speculated that this was due to the interaction of the peptide with the viral DNA, leading to the prevention of BrdU-antibody binding. This was further supported by the observation that the incubation of cells with TAT-I24 after fixation and permeabilization led to an impairment of BrdU-staining [16]. As additional support for this concept, BrdU-labelled MCMV was adsorbed to NIH/3T3 cells at 4 °C in the absence or presence of 10 µM TAT-I24. Cells were then washed with ice-cold medium followed by transfer to 37 °C and fixation and permeabilization 30 min post-cold release. In one setting, cells were additionally treated with pepsin for 20 min at 37 °C to degrade the peptide. While almost no BrdU-labelled genomes were seen in cells incubated with TAT-I24, post-fixation treatment of peptide-incubated cells with pepsin showed a clear appearance of a punctate pattern which was positively stained with the BrdU antibody (Figure 7). At high contrast settings, co-localization of BrdU- and 4´,6-diamidino-2-phenylindole (DAPI)-stained spots was observed (Figure 7), demonstrating the presence of viral DNA [16]. 

### 2.5. TAT-I24 Enhances the Antiviral Activity of the Nucleoside Analogue Cidofovir

**Figure 8:**
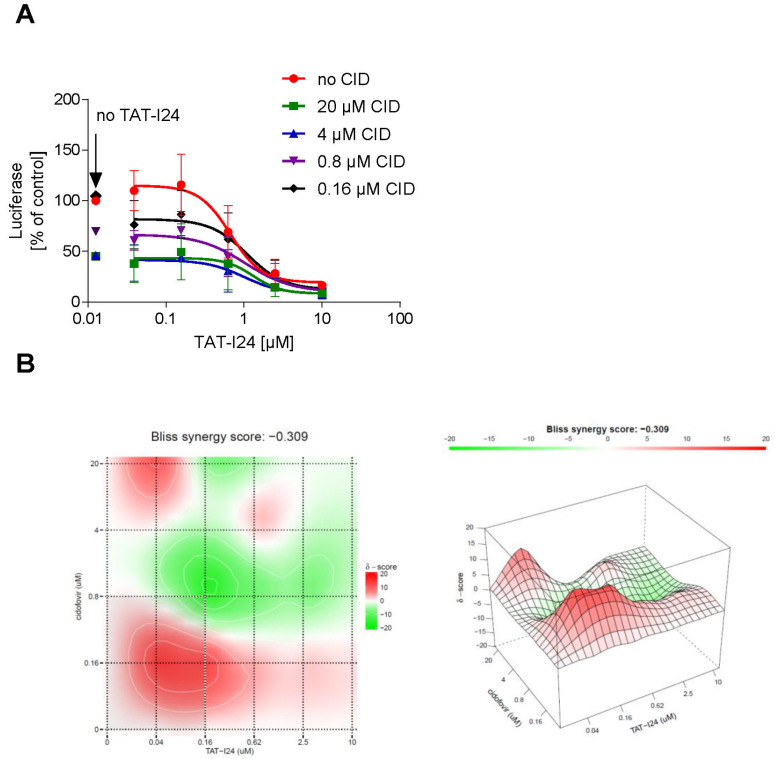
(**A**) Effect of a combination of TAT-I24 and cidofovir on replication of MCMV. Mean ± standard deviation of luciferase (expressed as % of untreated control) from cell lysates from three independent experiments performed in duplicates is shown. Luciferase levels in lysates of cells treated with various concentrations of cidofovir without TAT-I24 are indicated by an arrow “no TAT-I24”. (**B**) Bliss synergy plots of cidofovir and TAT-I24. Red areas indicate synergism while green areas indicate antagonism.

Based on the observation that the peptide can repress the expression of several viral genes, it could be an interesting candidate for use in combination with drugs like nucleoside analogues, which act at the stage of replication. The effect of a combined treatment of NIH/3T3 cells with TAT-I24 and cidofovir, a nucleoside analogue [37], on MCMV replication was therefore investigated. NIH/3T3 cells were infected with MCMV expressing luciferase and treated with various concentrations of cidofovir in combination with increasing concentrations of TAT-I24. After 72 h, luciferase was determined from cell lysates. Dose-response curves of the drug combinations showed an enhanced inhibitory effect of TAT-I24 when combined with cidofovir (Figure 8A). The SynergyFinder 2.0 software was used to calculate potential synergies using the Bliss independence model, which assumes that the two drugs act independently [38]. Positive synergy scores according to this model were calculated for the low concentration range (0.04 to 0.62 µM) of TAT-I24 combined with low concentrations of cidofovir and are indicated in red in the synergy plot. At higher concentrations of TAT-I24 and cidofovir, weak antagonistic effects were observed (Figure 8B).

## 3. Discussion

The present study demonstrates that after infection of NIH/3T3 cells with MCMV, the upregulation of several viral genes is repressed by TAT-I24. Inhibition is independent of whether these are immediate-early, early or late genes, as they were all reduced to a similar extent, thereby excluding a specific effect of the peptide on a particular viral gene. Based on earlier studies showing that TAT-I24 binds DNA with high affinity [16], together with the observed similarity in the suppression of immediate-early, early and late gene expression, it is likely that direct interactions with the viral DNA rather than the reduction of virus entry by the peptide account for the inhibition of viral gene expression. This is further supported by the observation that *ie1* transcript levels are more markedly reduced compared to the levels of viral DNA.

Host-genes, such as *Nfkbia*, *Ifit1*, *Cxcl10*, *Ccl7* and *Ccl5*, which contain binding sites for MCMV-activated transcription factors in their promoters [27,31,33], were also activated upon MCMV infection and their expression was suppressed or delayed by TAT-I24 only during the very early phase of infection (0.5–2 h post-infection). However, at later time-points, comparable levels of these transcripts were observed in both untreated and peptide-treated cells. It is possible that inhibition of expression of immediate-early genes, such as IE1, contributed to the suppression of host gene expression. The observation that reduced host-gene expression is dependent on peptides capable of binding to DNA with high affinity, which is also linked to their antiviral activity, would support such a dependence. However, downregulation of host genes such as *Ccl7*, *Ccl5* and *Cxcl10* by TAT-I24 at early time-points of infection could not be reversed when infection was performed with UV-irradiated MCMV, which was not able to induce viral gene expression due to severely damaged DNA [27]. This provides evidence for a direct effect of the peptide on host-gene expression rather than an indirect effect via downregulation of viral gene expression. In addition, reduction of virus entry is unlikely to be the cause of downregulation of host-gene expression, as this effect was only observed within the first hours of infection. Infection of cells with MCMV in the presence of peptide followed by treatment with pepsin further showed appearance of positive staining for BrdU. This provides additional evidence that peptide-treated cells were infected but the viral DNA was masked by the peptide.

The large double-stranded DNA genomes of HCMV and MCMV are recognized within infected cells by cytosolic DNA sensors such as cyclic guanosine monophosphate-adenosine monophosphate (GMP-AMP) synthase (cGAS), which activates the innate immune response via the cGAS-STING-TBK1-IRF3 pathway [39]. Masking of the viral DNA by the TAT-I24 peptide may also reduce its recognition by cytosolic sensors and could explain the transient reduction of virus-induced expression of host-genes, such as *Ifit1*, *Ccl7* and *Ccl5*. 

Together, these data show that the expression of host genes, which have been reported to be strongly upregulated upon infection with MCMV, is only affected at the early phases of infection, while the expression of viral genes is sustainably inhibited by TAT-I24, resulting in the impairment of productive virus generation in peptide-treated cells. This observation confirms previous findings that the peptide selectively affects viral gene expression by interaction with the viral genome and deepens our knowledge of the antiviral effect of this peptide [15,16].

The combination of TAT-I24 with a drug acting at a later stage of the viral life-cycle, such as a nucleoside analogue acting directly on DNA replication, showed an additive to synergistic inhibitory effect on MCMV replication at low concentrations of TAT-I24. The peptide could therefore be a potential candidate for the efficient inhibition of viral replication in combination with drugs acting at different stages of the viral life cycle. 

In summary, the present study demonstrates that TAT-I24 exerts its antiviral activity through selective inhibition of viral gene expression while only transiently affecting cellular genes, which are activated in response to the virus infection. However, it cannot be excluded that prolonged exposure of cells to the peptide could affect host-gene expression. This topic will be the focus of future studies to investigate the effect of prolonged treatment on gene expression of cells treated with TAT-I24.

## 4. Materials and Methods

### 4.1. Peptide and Compounds

The TAT-I24 (GRKKRRQRRRPPQCLAFYACFC) peptide was synthesized at Bachem AG (Bubendorf, Switzerland) and dissolved as 10 mM stock in DMSO. The peptides TAT (GRKKRRQRRRPPQ), TAT-M24 (RRRPPQCLAFYACFC), TAT-M25 (RRRCLAFYACFC) and TAT-M26 (GRKKRRCLAFYACFC) were synthesized at Proteogenix (Schiltigheim, France) and dissolved as 10 mM stock in DMSO. All peptide stocks were stored at –20 °C.

Cidofovir was purchased from Merck (Schnelldorf, Germany) and dissolved in water as 40 mM stock solution and frozen at –20 °C. Before addition to the cell culture medium, cidofovir was heated to 60 °C for a few minutes until the solution cleared.

### 4.2. Cell Culture 

NIH/3T3 cells were grown in CO_2_-independent medium supplemented with 10% fetal calf serum, 2 mM glutamine and 1% antibiotic-antimycotic (ThermoFisher; Darmstadt, Germany) and cultivated in a humidified atmosphere at 37 °C and passaged once a week. For determination of cell viability, NIH/3T3 cells were seeded at a density of 2 × 10^4^ cells/well of a 96-well plate and treated with vehicle (DMSO) or 10 µM TAT-I24. After 48 h, cells were lysed using Cell Titer Glo 2.0 reagent (Promega; Mannheim, Germany) and luminescence recorded using a GloMax Multi instrument (Promega; Mannheim, Germany). 

### 4.3. Murine Cytomegalovirus

NIH/3T3 cells were seeded at a density of 8 × 10^4^ cells/well of a 24-well plate and allowed to attach overnight. On the next day, MCMV strain delm157-luc rep [40] was adsorbed to cells (MOI 1) in the absence or presence of 10 µM TAT-I24 and centrifuged twice at 800× *g* for 15 min. Cells were then kept at 37 °C and lysed after the indicated time-points. 

For virus inactivation by ultraviolet (UV) irradiation, MCMV stock was exposed to UV-light in a transilluminator (312 nm) and covered with aluminum foil for 10 min adapted from Watanabe et al. [41].

### 4.4. RNA Isolation, cDNA Synthesis and Quantitative Real-Time PCR

RNA was isolated using RNeasy Mini kit (Qiagen; Hilden, Germany) and eluted with 40 µL of nuclease-free water. To remove viral DNA, 10 µL of RNA extracts were treated for 30 min with DNase I (Qiagen; Hilden, Germany) in a 20 µL reaction volume before subjection to cDNA synthesis (9 µL RNA/reaction) using High-Capacity cDNA Reverse Transcription Kit (Applied Biosystems/ThermoFisher; Darmstadt, Germany). cDNA was then diluted to 60 µL with nuclease-free water and stored at −20 °C.

All primers were synthesized at Microsynth AG (Balgach, Switzerland). PCR primers for detection of the viral genes *m123/ie1*, *m152*, *m169*, *m129/m131* and *M94*, as well as primers for detection *NFkbia*, *Ifit1*, *Herpud1*, *Top2a* and *Gadd45a* were synthesized according to the sequences published by Krause et al. [31] and Marcinowski et al. [27]. Additional primers used in this study were: *Cxcl10* forward: 5’-TCTGAGTGGGACTCAAGGGAT–3’ and *Cxcl10* reverse: 5’-ATTCTCACTGGCCCGTCATC–3’; *Ccl7* forward: 5’-CCCTGGGAAGCTGTTATCTTCAA–3’ and *Ccl7* reverse: 5’-CTCGACCCACTTCTGATGGG–3’; *Ccl5* forward: 5’-CACCATATGGCTCGGACACC-3’ and *Ccl5* reverse: 5’-CCTTCGAGTGACAAACACGA-3’; *Gapdh* forward: 5’-CTCCCACTCTTCCACCTTCG–3’, and *Gapdh* reverse: 5’-GCCTCTCTTGCTCAGTGTCC–3’. 

For PCR, 2 µL cDNA was added to a reaction mix containing Luna^®^ Universal qPCR Mastermix (New England BioLabs, Frankfurt am Main, Germany) and 250 nM of each gene-specific primer in a 20 µL reaction mix using a QuantStudio™ 7 Flex Real-Time PCR System (ThermoFisher, Darmstadt, Germany). Transcript levels relative to the *Gapdh* housekeeping gene were calculated according to the 2^−^^ΔΔCT^ method [42]. To exclude the presence of residual viral DNA, DNAse I-digested RNA was subjected to *ie1* PCR without reverse transcription. In addition, viral DNA was amplified from 1 µL undigested RNA extracts using the *ie1* primer pair. 

### 4.5. Luciferase Assay

NIH/3T3 cells were seeded at a density of 2 × 10^4^ cells/well of a 96-well plate and allowed to attach overnight. On the next day, cells were treated with various concentrations of peptides, or cidofovir either alone or in combination with increasing concentrations of TAT-I24 and infected with MCMV strain delm157-luc rep [40] with centrifugal enhancement twice at 800× *g* for 15 min. After 72 h, cells were lysed and luciferase measured using luciferase assay system (Promega; Mannheim, Germany) using a GloMax Multi instrument (Promega; Mannheim, Germany). 

### 4.6. DNA Binding Assay

Binding of peptides to double-stranded DNA was determined by reduction of fluorescence from SYBR^®^ Gold-stained plasmid DNA as described previously [16].

### 4.7. Microscopy

NIH/3T3 cells were seeded at a density of 2 × 10^4^ cells/well of a 96-well plate and allowed to attach overnight. On the next day, cells were treated with increasing concentrations of TAT-I24 and infected with MCMV strain delm157-luc rep with centrifugal enhancement twice at 800× *g* for 15 min. After 48 h, cells were fixed with 4% formaldehyde and stained with 1% crystal violet solution for 30 min at room temperature followed by three times washing with water. Microscopic examination was performed using a Live Cell Video Microscope (Leica Microsystems; Wetzlar, Germany).

NIH/3T3 cells were seeded at a density of 4 × 10^4^ cells/well into ibiTreat eight-well chambers (ibidi, Gräfelfing, Germany). Bromodeoxyuridine (BrdU)-labelled virus was adsorbed to cells in the absence or presence of 10 µM TAT-I24 as described before [16]. Pepsin treatment was performed by incubation of cells after fixation, permeabilization and denaturation with Pepsin Reagent, Ready to Use, Antigen Retriever (Merck; Schnelldorf, Germany) for 20 min. Cells were then washed twice with 1xTBE followed by three washes with PBS. Staining with BrdU-antibody was performed as described earlier [16].

### 4.8. Statistics

Curve fittings and statistics were calculated using GraphPad Prism 8 (GraphPad Software, San Diego, CA, USA). Bliss synergy scores were calculated using SynergyFinder 2.0 software (https://synergyfinder.fimm.fi (accessed on 25 June 2022)) [38].

## 5. Patents

Hanna Harant is the inventor of patent application WO2019/057973 “Gene expression inhibitors”.

## Data Availability

Not applicable.
